# Innovative technology for evaluation of sperm DNA double-strand breaks diagnoses male factor infertility and prevents reproductive failures

**DOI:** 10.1038/s41598-023-46049-4

**Published:** 2023-11-03

**Authors:** Tse-En Wang, Chun-I. Lee, Chun-Chia Huang, Hui-Mei Tsao, Hui-Chen Chang, Li-Sheng Chang, T. Arthur Chang, Maw-Sheng Lee, Cheng-Teng Hsu

**Affiliations:** 1 Center for Research and Development, Bonraybio Co., Ltd., 4F., No. 118, Gongye 9Th Rd., Dali Dist., Taichung City, 412037 Taiwan, ROC; 2 Division of Infertility Clinic, Lee Women’s Hospital, No. 30-6, Sec. 1, Changping Rd., Beitun Dist., Taichung City, 406021 Taiwan, ROC; 3 Institute of Medicine, Chung Shan Medical University, Taichung, Taiwan, ROC; 4 Department of Obstetrics and Gynecology, Chung Shan Medical University, Taichung, Taiwan, ROC; 5 Department of Post-Baccalaureate Medicine, National Chung Hsing University, Taichung, Taiwan, ROC; 6 Department of Obstetrics and Gynecology, University of Texas Health Science Center, San Antonio, TX USA

**Keywords:** Biotechnology, Health care, Molecular medicine, Risk factors, Urology

## Abstract

Neutral comet assay has been available for two decades to evaluate sperm double-strand breaks (DSBs). However, its clinical usability is limited due to its complex and time-consuming procedure, as well as the lack of a standardized scoring system. The aim of this study was to: develop a rapid diagnostic method for DSBs, Sperm DNA Fragmentation Releasing Assay (SDFR), and explore the association between DSBs and reproductive outcomes. We pioneered the use of polyacrylamide (PA) for embedding sperm chromatin and optimized the porosity of PA to be between 10 and 13%. The refined PA network allowed the trapping of DSBs, which dispersed halo on an immunological slide; in contrast, intact chromatin failed to develop a halo. A strong correlation was showed between reproducible values obtained from SDFR and neutral comet assay. SDFR were responsive to dose-/time-dependent simulated DSBs, indicating high sensitivity and specificity. Furthermore, we conducted a retrospective study of couples with embryonic aneuploidy screening, and recording DSB profiles of the male partners. Our findings revealed that DSB enabled to predict embryonic aneuploidy whereas basic semen parameters did not. In conclusion, SDFR offers a rapid and user-friendly approach for evaluating DSBs, with potential implications for predictive healthcare in reproductive medicine.

## Introduction

Approximately 48.5 million couples suffer from infertility worldwide, with male factors accounting for about 20–30% of all infertility cases^1^. Basic semen examination covers parameters such as semen volume, sperm concentration, motility, and morphology^3^. However, up to 40% of male-infertility cases display no identified abnormalities in basic semen parameters^4^. A meta-analysis on the probability and indication of sperm DNA defects showed that sperm DNA fragmentation (SDF) is a primary diagnostic factor in these idiopathic infertility cases. Moreover, SDF is linked to reproductive failures, including lower fertilization rate^5^ and clinical pregnancy^6^.

The WHO 6^th^ edition guideline outlined four standard methods for assessing SDF: (1) TdT (terminal deoxynucleotidyl transferase)-mediated dUTP nick-end labeling (TUNEL), (2) sperm chromatin dispersion (SCD), (3) sperm chromatin structure assay (SCSA), and (4) comet assay^5,9^. Much attention was given to enhance the reliability of SDF assessment through the integration of fluorescent amplification detection systems^6^ or high-throughput flow cytometry^10^. However, the mentioned methods quantify “total” SDF but do not enable the differentiation between single-strand breaks (SSBs) and double-strand breaks (DSBs) in DNA fragments.

Considering DSBs are significantly more lethal than SSBs because they are harder to repair^11,12^. An early study developed an alternative comet assay by performing the comet assay under neutral conditions^13^. Neutral comet assay relies on the unique behavior of DSBs under neutral non-denaturing conditions, whereby stretches of DNA molecules between the DSBs physically detach and diffuse from the nucleoid. With the aid of electrophoresis, the detached DSBs migrate to the anode end and form a comet tail. Neutral comet assay has attracted attention due to its ability to exclusively correlate with DSBs in clinical settings. High DSB profile have been pathologically associated with recurrent pregnancy loss^14,15^, delayed embryo development, and impaired implantation rates^16^, links that were barely found using conventional methods. However, neutral comet assay has been criticized for being a laborious and time-consuming procedure, with a subjective and unstandardized criterion for DFI quantification, which significantly limits its usability in clinical applications.

In response to the growing need for a time-saving and easy-to-perform method for evaluating DSBs, researchers have explored another molecular property of DSBs. These breaks are preferentially located at matrix attachment regions (MAR regions) of sperm chromatin, where endonucleases actively participate. The endonuclease-dependent DSBs result in specific fragment sizes, typically around 50 kb^14,17^. The aim of this study was to: (1) develop an innovative technology for DSB evaluation based on their unique molecular characteristics, (2) validate its reliability, sensitivity, and specificity, and (3) use it to explore the clinical association of DSBs with reproductive outcomes.

## Materials and methods

### Ethics statement

The prospective cohort study was approved by the Institutional Review Board of Chung Shan Medical University and was registered with the reference number CS2-20012. All patients were fully counseled and provided informed written consent before entering the study. The study protocol was designed and validated in accordance with the Declaration of Helsinki.

### Participants enrollment

We recruited a total of 640 infertile men who attended Lee Women’s Hospital (Taichung, Taiwan) seeking assisted reproductive treatments from May 2020 to December 2021. Male patients of couples seeking fertility treatment were eligible. The main selection criteria were age 20–60 years old and signed consent for participation in the study. Exclusion criteria in the prospective cohort included patients with azoospermia, and/or retrograde ejaculation.

### Basic semen analysis

Semen samples were collected by masturbation into sterile cups following 2–5 days of abstinence. After full liquefaction, aliquots of the semen were loaded into a 10 µm deep chamber slide and analyzed using a CEROS II device (Hamilton-Thorne, Danvers, MA) for sperm concentration, total motility, and progressive motility.

### Assessment of total DFI

Total DFI was conducted by LensHooke® R10 Sperm Chromatin Dispersion Assay (Bonraybio, Taichung, Taiwan), according to the manufacturer’s instructions^18^. Briefly, a tube containing agarose was heated at 100 °C for 5 min and subsequently cooled down at 37 °C. A semen aliquot of 25 μL (10 × 10^6^/mL) and 25 μL denaturant solution were added to the tube, and a 25 μL admixture was placed on a pretreated microscope slide and covered with a 22 × 22 mm coverslip. The slide underwent a lysis reaction at room temperature (RT) for 10 min. The slide was washed with distilled water for 5 min and dehydrated in 95% methanol for 1 min. The slide was stained with Wright-Giemsa solution. A large and/or medium halo were referred to intact sperm DNA and small and/or no halo were fragmented DNA. Total DFI was interpreted under a bright-field microscope (Olympus BX53). At least 500 sperm cells were examined per test.

### Assessment of DSB DFI

#### LensHooke® R11 sperm DNA fragmentation releasing assay

DSB DFI was performed by LensHooke® R11 Sperm DNA Fragmentation Releasing Assay (Bonraybio). A semen aliquot of 70 μL was mixed with 70 μL 30% (w/v) acrylamide/bis-acrylamide solution (BioRad, Hercules, CA), 15 μL 1% (w/v) ammonium persulfate (BioRad), and 15 μL tetramethylethylenediamine (TEMED, BioRad) to initiate gel polymerization in a 1.5 mL microcentrifuge. A 15 μL aliquot of the mixture was immediately placed onto the pretreated microscope slide and covered by a 24 × 40 mm coverslip. The slide was horizontally placed at RT for 5 min and the coverslip was carefully removed. The lysis solution (0.4 M Tris, 1 M Urea, 0.05% SDS, 50 mM TCEP, 50 mM Na_2_EDTA, 2.5 M NaCl, 1% Triton X-100, and 5 mM NaOH, pH 8.0) was immediately added to the slide and the slide was incubated at RT for 10 min, followed by tilting to drain off the residual reagents. The slide was immersed in distilled water for 5 min, Diff-Quik I for 1 min, Diff Quik II for 1 min, and de-stained with 75% ethanol for 1 min. The dried slide was evaluated under a bright-field microscope. Sperm with a large and/or medium halo were referred to as sperm with DSBs. DSB DFI was calculated as the percentage of spermatozoa with a halo over the total population. A minimum of 500 spermatozoa were scored per test sample.

#### Neutral comet assay

A semen aliquot of 25 μL (2 × 10^6^/mL) was mixed with 50 μL 1% low melting point agarose. Next, 20 μL sperm–agarose mixture was placed on a Cometslide™ (Trevigen®) for gel adhesion, covered with a coverslip, and solidified at 4 °C for 5 min. The coverslip was gently removed and the slide was incubated with lysis solution 1 (0.8 M Tris–HCl, 0.8 M DTT, 1% SDS, pH 7.5) followed by lysis solution 2 (0.4 M Tris–HCl, 50 mM EDTA, 2 M NaCl, 0.4 M DTT, pH 7.5) for 30 min at RT each. The slide was washed in TBE (0.4 M Tris–HCl, 0.4 M boric acid, 10 mM EDTA) buffer for 10 min. and placed in an electrophoresis tank with freshly prepared TBE buffer. Electrophoresis was performed at 20 V (1 V/cm), 12 mA for 12.5 min. After electrophoresis, the slide was washed with 0.9% NaCl and dehydrated in an ethanol series (70, 90, and 100%) for 2 min each. Finally, the Cometslide was stained with 1 × SYBR™ Green I (Thermo Fisher Scientific Inc) and images were captured using a digital camera imaging system U3CMOS05100kpa and ToupView software (ToupTek Photonics, China), incorporated to an epifluorescence microscope (Olympus BX53). Sperm comet assay images on the captured microphotographs were analyzed using the CometScore 2.0 software (TriTek, Sumerduck, VA). Counting at least 300 sperm cells per sample. Sperm were defined as DSBs following criteria of the length of DNA tail being ≥ 40 µm and having a percentage of the DNA tail over total signal ≥ 20%.

### Evaluation of R11 sensitivity and specificity

#### Time/dose-dependent treatment of DNase I and Alu I

DNase I and Alu I are endonucleases commonly used to simulate DSBs^14,19^. Accordingly, R11 sample slides were treated with 40 U/mL of deoxyribonuclease I (DNase I) (Zymo Research Corporation, Irvine, CA), prior to the lysis, for 5, 10, 15, and 30 min at 37 °C; alternatively, they were treated with 0, 5, 10, 20, and 40 U/mL DNase I at 37 °C for 30 min to simulate the different degrees of DSBs. Similarly, R11 sample slides were treated with 200 μL 50 U/mL Alu I (New England Biolabs, Ipswich, MA) for 10, 20, 30, and 40 min at 37 °C, while the sample slides were treated with 5, 15, 25, and 50 U/mL Alu I at 37 °C for 30 min. Hereafter, the slides were washed with distilled water and the same procedure as the R11 general protocol, as described above, was followed.

#### Time/dose-dependent treatment of hydrogen peroxide

Hydrogen peroxide (H_2_O_2_) can induce oxidative stress, which predominantly causes SSBs^20^. The semen samples were incubated with 0.03, 0.15, and 0.3% H_2_O_2_ (Sigma Aldrich, St Louis, MO, USA) in PBS at RT for 1 h to generate different SSB levels. After the treatment, semen samples were washed and resuspended in PBS buffer to the proper concentration (10 × 10^6^/mL). The H_2_O_2_-pretreated semen aliquots were analyzed by R10 and R11, as described above.

### Preimplantation genetic testing for embryonic aneuploidy

In the cohort of this study, 140 couples had two or more embryos undergoing preimplantation genetic testing for aneuploidy (PGT-A) using VeriSeq-NGS at Lee Women’s Hospital. Embryos were classified as aneuploidy if the level of mosaicism was greater than 80% based on the consensus established by international genetic testing community; aneuploidy rates were calculated per number of biopsied blastocysts^21^.

### Statistical analysis

After testing for normal distribution using the Shapiro–Wilk test, the continuous variables with normal distribution were presented as means ± standard deviations (SD), and comparative analysis was performed by using one-way ANOVA. The results with skewed distributions were presented as medians ± interquartile ranges and the Kruskal–Wallis test was applied for pairing analysis. A comparison of DSB DFI measured by R11 and neutral comet assay was performed by Bland–Altman plot analysis. Spearman correlation analysis was used to assess the association between aneuploidy rate and semen parameters. The area under the curve (AUC) was calculated to determine the performance of the prediction model in discriminating aneuploidy from the controls. A p-value less than 0.05 was considered statistically significant. Statistical analysis was performed by Prism software, version 6.01 (GraphPad Software, Inc, San Diego, CA, USA).

## Results

### Determining the proper concentration of polyacrylamide for DSB detection

**Figure 1:**
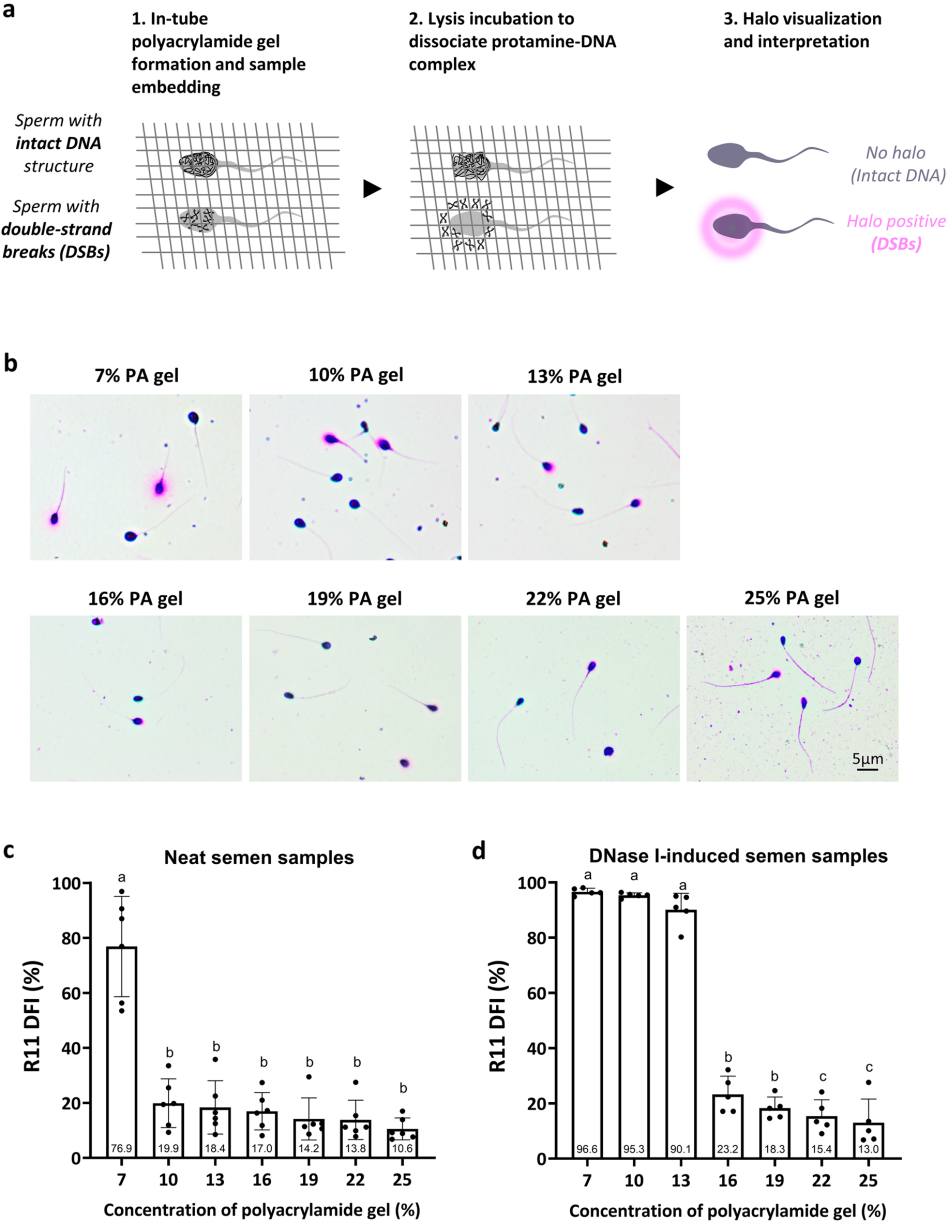
Determining the proper concentration of polyacrylamide for sperm DSB evaluation. (**a**) A conceptual schema of R11 technique for DSB evaluation. (**b**) Representative images derived from normozoospermic semen samples analyzed by R11 under different concentrations of polyacrylamide (PA). Quantitative results of DFIs derived from (**c**) normozoospermia semen samples, and from (**d**) DNase I treated semen samples. Different alphabets indicate a significant difference between two methods (*p* < 0.05).

The conceptual model of R11 is illustrated in Fig. 1a. By optimizing the polyacrylamide (PA) microgel porosity the DSBs are released and then trapped within the PA network (halo positive). In contrast, due to the feature of the convoluted chromatin structure, the chromatin of the healthy sperm is spatially restricted and fails to disperse to the halo (no halo).

Normozoospermia samples (n = 5) were split into 7 aliquots and each aliquot was embedded into a 7, 10, 13, 16, 19, 22, and 25% PA gel. Quantitative R11 DFI results were no difference between 10 and 25% PA microgels whereas the value significantly increased when using 7% PA (76.9%, *p* < 0.05) (Fig. 1c). The illustrations of halo were shown in Fig. 1b. In the validation using DNase I treatment, no difference in R11 DFI was observed between 7 and 13% PA groups. However, a significantly lower level of R11 DFI was detected in the 16%, 19%, 22%, and 25% PA groups (*p* < 0.05) (Fig. 1d). Collectively, 7% PA was too large to trap DSBs, leading to false positive results. Conversely, PA concentration above 13% were too small to disperse the DSBs, resulting in false negative results. Therefore, a PA concentration of 12% was used for further evaluation.

### Correlation of DFI between R11 and neutral comet assay

**Figure 2:**
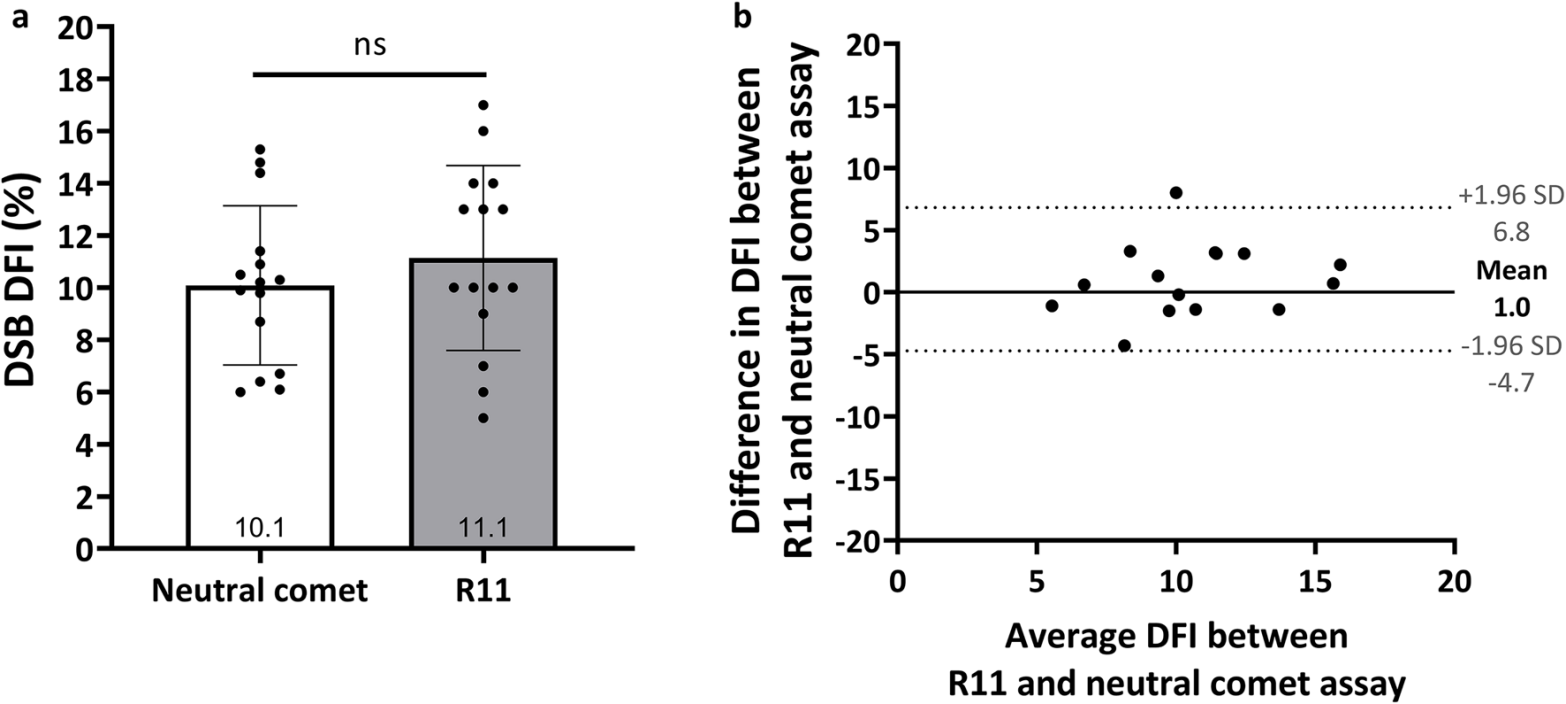
Correlation of DFI between R11 and neutral comet assay. (**a**) Aliquots of semen samples were evaluated by neutral comet assay and R11 assay. (**b**) Bland–Altman analysis of DFI deviation between neutral comet and R11 assays. The solid black line represents the mean of the two assays and the black dashed lines are the 95% confidence ranges.

Liquefied semen samples from 15 men were analyzed by R11 and neutral comet assay. There was no significant difference in DSB DFI between the neutral comet (mean ± SD: 10.1 ± 3.1), and R11 assays (mean ± SD: 11.1 ± 3.5) (Fig. 2a). The Bland–Altman plot analysis showed a mean bias of 1.0% and the 95% limit agreement ranged from 6.8% to -4.7%, presenting a good agreement between the two assays (Fig. 2b).

### R11 sensitivity and specificity evaluation

**Figure 3:**
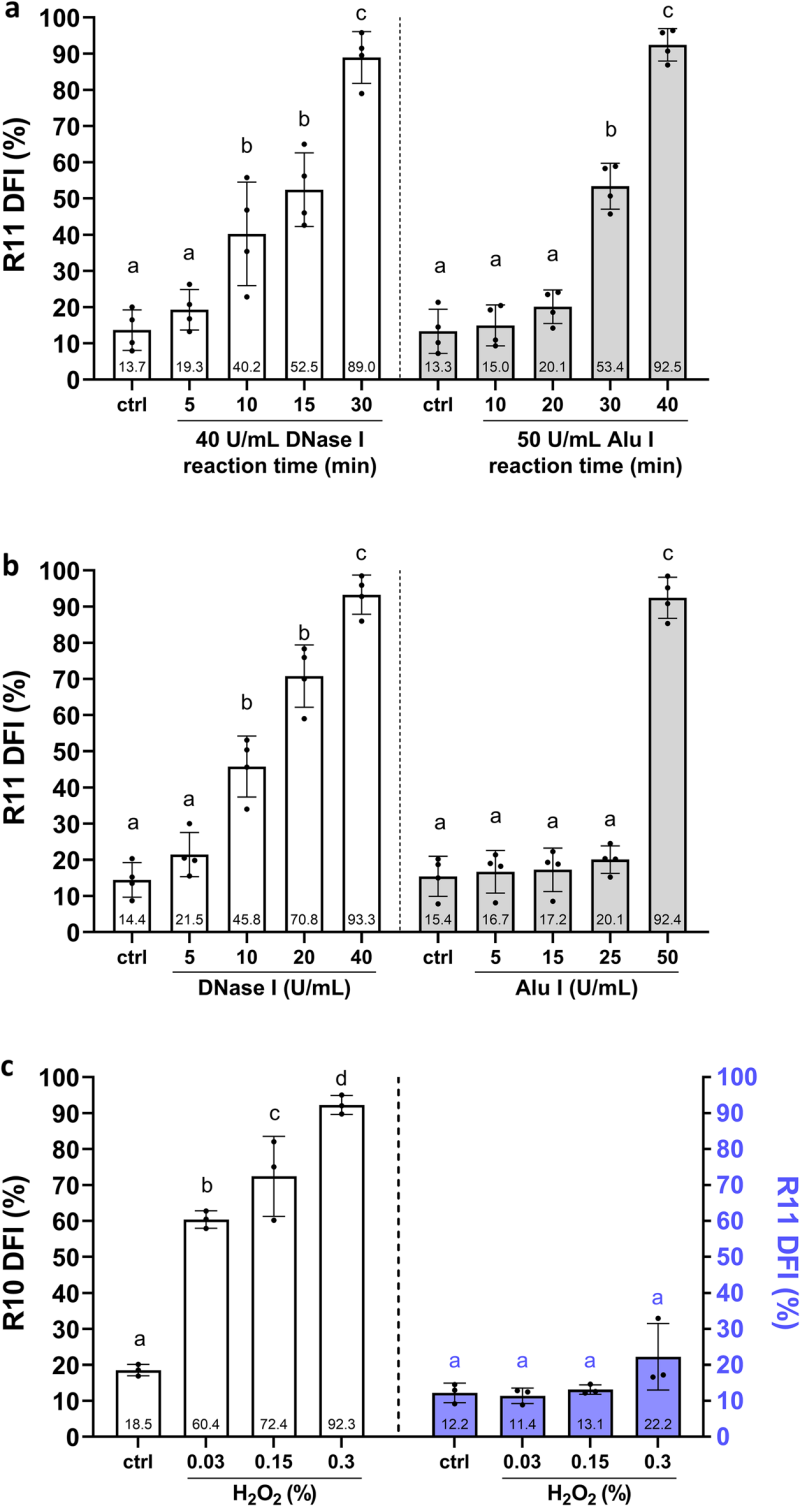
Sensitivity and specificity for detecting DSBs using R11 assay. (**a**) DFI responses for DSBs with different incubation times of DNase I and Alu I. (**b**) DFI responses for DSBs with different dosages of DNase I and Alu I treatment. (**c**) H_2_O_2_-induced semen samples were analyzed by R10 for total DFI evaluation, and R11 for DSB DFI evaluation. Different alphabets indicate a significant difference between the two methods (*p* < 0.05).

By performing time-dependent and dose-dependent enzymatic treatments of DNase I and Alu I, R11 DFI increased alongside the extension of the treatment time and doses (Fig. 3a,b). Conversely, we induced SSBs by H_2_O_2_ treatments, and the pretreated specimens were analyzed for the total DFI by R10 and for DSB DFI by R11. Figure 3c shows that the R10 DFI increased dose-dependently with the higher H_2_O_2_ concentrations. In contrast, the R11 DFI remained at basal levels regardless of H_2_O_2_ concentration. Therefore, R11 can specifically detect sperm DSBs.

### Significant correlation of R11 DFI with basic semen parameters

R11 DFI negatively correlated with total motility (Spearman *r* = -0.2732, *p* < 0.0001), progressive motility (Spearman *r* = -0.1236, *p* = 0.0027), and normal morphology (Spearman *r* = -0.1870, *p* < 0.0001). The DFI was positively correlated with the age of the male (Spearman *r* = 0.3694, *p* < 0.0001) and R10 DFI (Spearman *r* = 0.8054, *p* < 0.0001).

### Clinical association of R11 DFI with embryonic aneuploidy

Among five semen parameters: sperm concentration, total motility, normal morphology, R10 (total DFI), and R11 (DSB DFI), R11 (AUC = 0.66, *p* = 0.0007) possessed value in predicting the prevalence of aneuploidy (high aneuploidy rate ≧ 50%). Given that parental age: female age (AUC = 0.70, *p* < 0.0001) and male age (AUC = 0.67, *p* = 0.0002) were also indicated to have clinical relevance with embryonic aneuploidy (Fig. 5a; Supplementary Table S2 online), we performed an adjusted model that excluded advanced female ages (female age > 38-years-old) to account for any confounding factors. The results showed that R11 had the highest value in predicting aneuploidy (AUC = 0.68, *p* = 0.002) compared to the other semen quality indexes. The R11 threshold was > 8% and the clinical sensitivity and specificity reached the best balance (Fig. 5b; Supplementary Table S3 online).

## Discussion

Herein, we demonstrated that LensHooke® R11 rapid assay detects sperm DSBs with high sensitivity and high specificity. R11 opens a new spectrum for male infertility diagnoses due to the identification of distinct sperm DNA damage profiles.

Agarose is commonly used as the materials for sperm chromatin embedding in the conventional methods such as sperm chromatin dispersion (SCD) and comet assays. In the development of R11, we introduced the use of polyacrylamide (PA) for the first time. PA possesses higher molecular complexity than agarose. By optimized porosity at 10–13% PA microgel, we demonstrated that DSBs are effectively trapped within the PA network and the quantitative results are consistent with neutral comet assay (Figs. 1, 2). Attributed to the innovative application of PA and electrophoresis-free procedure, R11 provides a time-saving protocol compared to the neutral comet assay. The average operation of the neutral comet assay is 141.5 min, while it is only 35 min for R11 (Supplementary Fig. 1 online, Supplementary Table S1 online).

Furthermore, R11 has advantages over neutral comet assay in the standardization process of DFI interpretation. Scoring of the comets can be performed with the help of analytic software to reduce inter-observer/technician variability^22,23^; however, diverse formulae for fragmented DNA calculation exists between laboratories, such as percent DNA in the tail (%), tail length (μm), and tail moment (%)^24^. This diverse standardization leads to high intra-laboratory variability and no unanimous consensus on a specific cut-off value for clinical reference. Herein, R11 offers a standard binary classification of “halo” for DSBs and “no halo” for intact DNA that minimizes the subjectivity of DFI interpretation. Furthermore, the image-based results have the potential to conjugate with artificial intelligence (AI)-integrated automated system^18^, thereby making DSB evaluations faster, standardized, and laboratory manageable.

**Figure 4:**
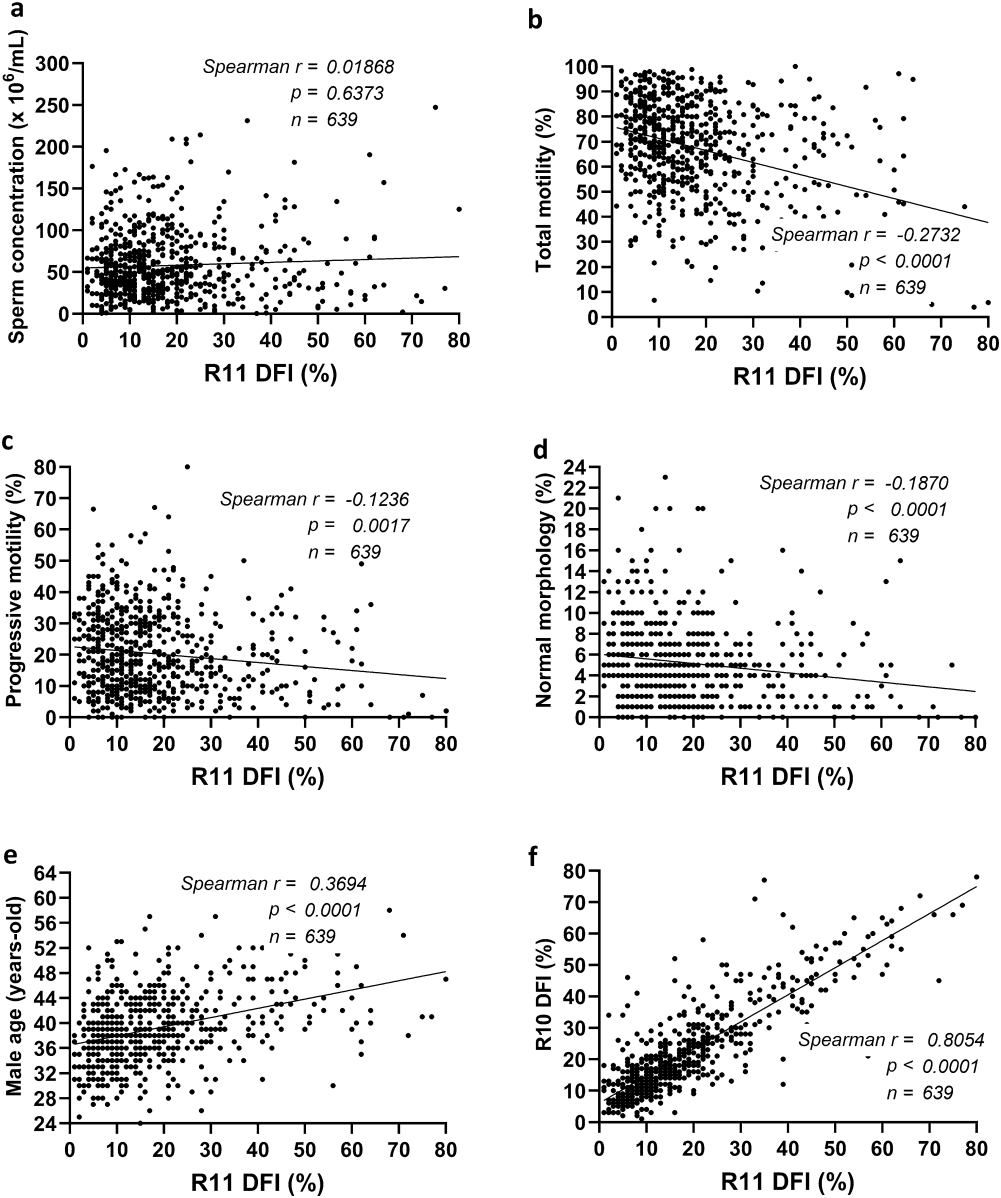
Correlation of R11 with semen parameters. A scatter and linear regression plot comparing R11 DNA fragmentation index (DFI, %) versus (**a**) sperm concentration (× 10^6^/mL), (**b**) total motility (%), (**c**) progressive motility (%), (**d**) normal morphology (%), (**e**) male age (years-old), and (**f**) R10 DFI (%).

**Figure 5:**
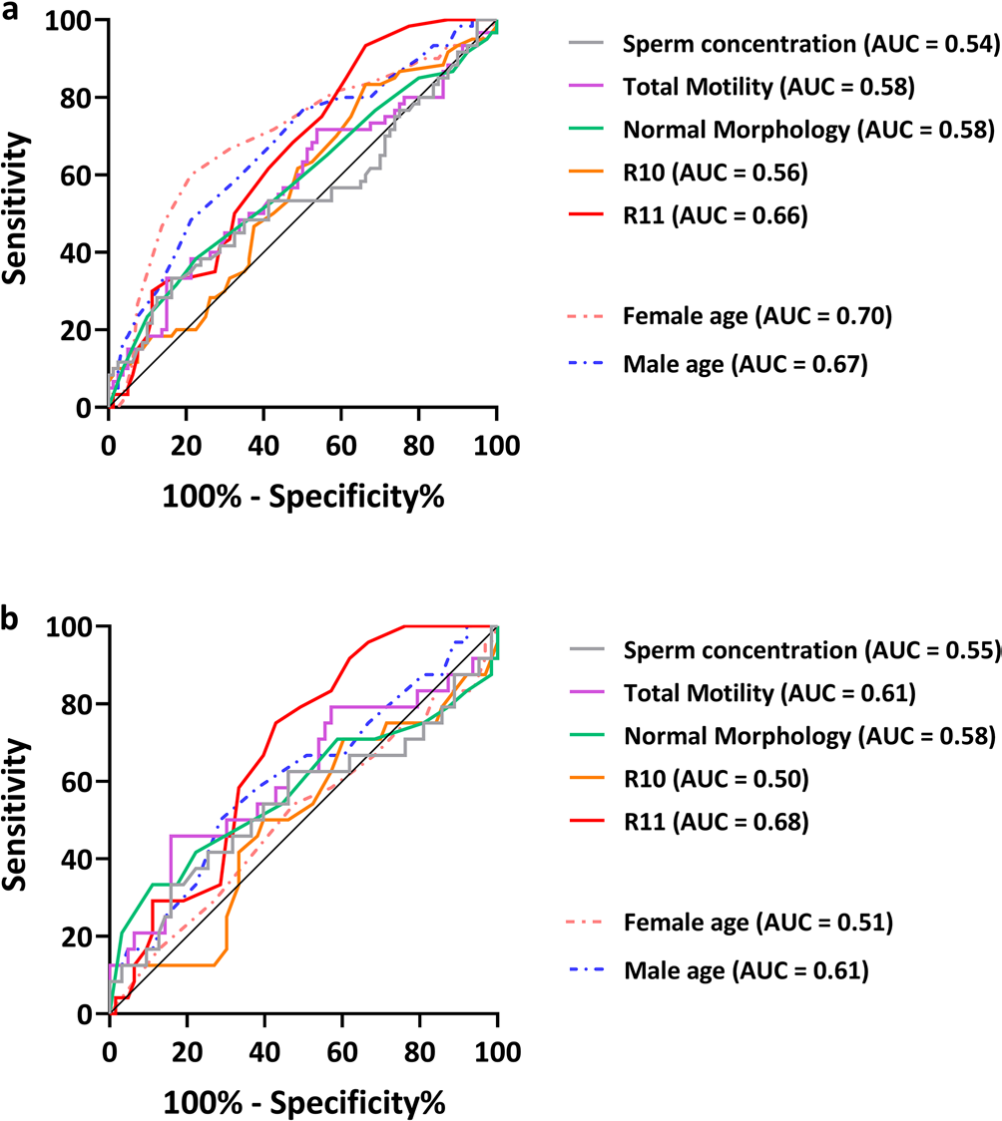
The ROC curves for differentiating sperm samples between low aneuploidy rate and high aneuploidy rate. (**a**) Unadjusted model for advanced female age comprised 60 cases with an aneuploidy rate ≧ 50% (high), and 80 cases with an aneuploidy rate < 50% (low). (**b**) Adjusted model, excluding cases where female partners were > 38-years-old, comprising 24 cases for high aneuploidy rate, and 63 cases for low aneuploidy rate.

In the present study, we proved R11 sensitivity and specificity using simulated samples (Fig. 3) and also explored the feasibility of the method in biological samples. In a cohort of 640 male patients, R11 showed a significant correlation with total motility, progressive motility, and morphology (Fig. 4), which was in accordance with previous findings^6,10,25^. However, notwithstanding, sperm DSBs are known to associate with recurrent pregnancy loss; thus, the outcomes of increased DSB were used as a retrospective indicator for patients who have suffered failure(s) of natural conception and/or assisted reproduction attempts^14,17^. Here, we demonstrated the significant predictive power of R11 in identifying preimplantation embryonic aneuploidy, even in a model that excludes confounding variables related to advanced female age^25^ (Fig. 5b). Therefore, sperm DSB evaluation can be considered as a primary approach for assessing paternal risk factors and mitigating the impacts associated with DSBs. On the other hand, high probability of embryonic aneuploidy was exclusively identified in the profile of DSBs using R11 but not in total DFI using R10. The findings align with previous studies that compared DSBs profile using neutral comet assay and total DFI using alkaline comet assay^14,15,26^. It is indicated that the evaluation of total DFI alone may not fully represent the status of sperm chromatin. The combination of these two SDF indexes provides greater sensitivity in understanding the etiology of male factor infertility, which can further assist clinicians in determining treatment strategies. Nevertheless, the limitations of the current study are single-center trial, and small sample size; more clinical trials are needed to solidify the relationship between R11 and ART failures.

In conclusion, we developed a rapid assay for evaluating sperm DSBs, which does not require electrophoresis and has standard criteria for DFI interpretation; these advantages are beneficial to improve the effectiveness of semen analysis in clinical practice. Additionally, R11 is dedicated to early diagnosis of embryonic aneuploidy that can facilitate treatment plan decision-making workflows and reduce the financial and emotional burdens for ART couples.

### Supplementary Information

Supplementary Figure 1.

Supplementary Table 1.

Supplementary Table 2.

Supplementary Table 3.

## Data Availability

The data used and/or analyzed during the current study available from the corresponding author on reasonable request. The corresponding author has access to all the data and takes responsibility for the integrity of the data and accuracy of the data analysis.
