# Supplementary material for: Innovative technology for evaluation of sperm DNA double-strand breaks diagnoses male factor infertility and prevents reproductive failures
Source: Sci Rep. 2023 Nov 3;13:18996. doi: 10.1038/s41598-023-46049-4 (PMC10624885; doi:10.1038/s41598-023-46049-4)
Supplement: Supplementary file 1 — Supplementary Figure 1. [file 41598_2023_46049_MOESM1_ESM.docx]

**Supplementary Figure S1. Comparison of DFI interpretation process between neutral comet and R11 assays.**

**
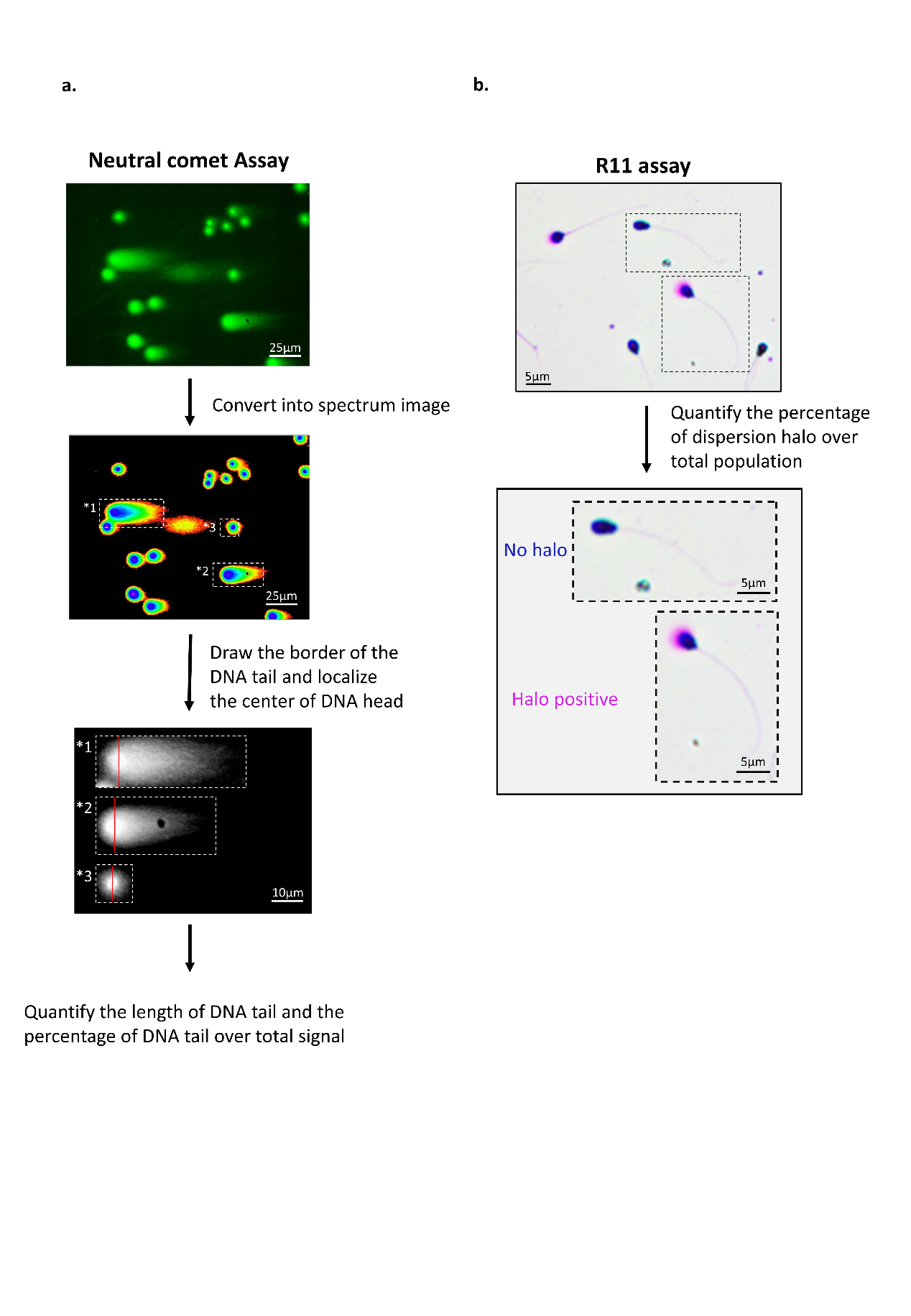
**

Illustrate the workflow of the DFI quantification using neutral comet assay **(a),** and R11 assay **(b)**. The scoring system of neutral comet assay involves converting the fluorescent images into a spectrum view, manually localizing the DNA head, tail, and center, and measuring the DNA tail length and the percentage of DNA tail over the total signal. One-step quantification of R11 is performed by identifying halo-positive sperm under a bright-field microscope. *Abbreviation: DFI (DNA fragmentation index)
