# Supplementary material for: Innovative technology for evaluation of sperm DNA double-strand breaks diagnoses male factor infertility and prevents reproductive failures
Source: Sci Rep. 2023 Nov 3;13:18996. doi: 10.1038/s41598-023-46049-4 (PMC10624885; doi:10.1038/s41598-023-46049-4)
Supplement: Supplementary file 2 — Supplementary Table 1. [file 41598_2023_46049_MOESM2_ESM.docx]

**
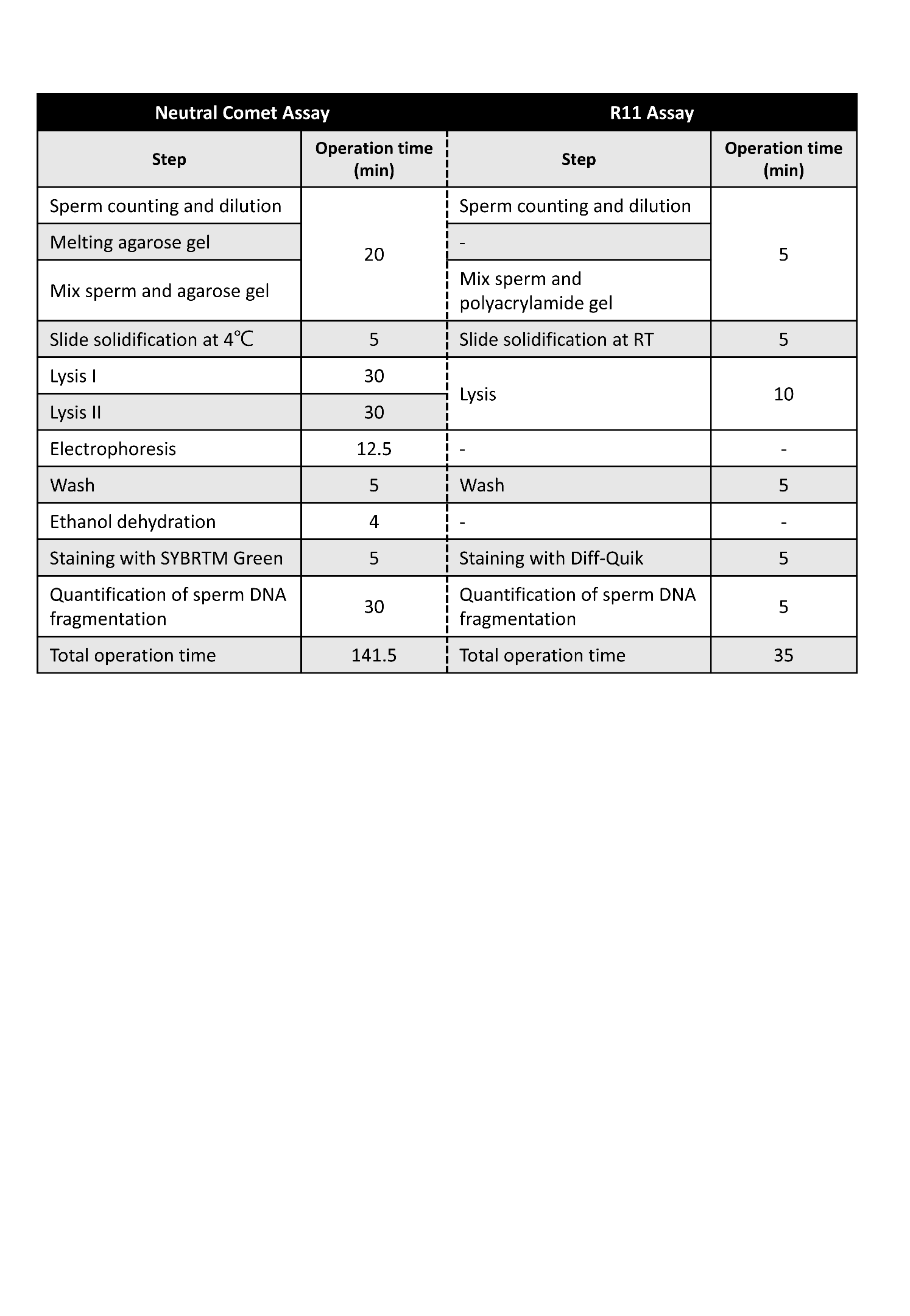
Supplementary Table S1. Comparison of protocol and assay time between using neutral comet assay and R11 assay.**
