# Supplementary material for: Innovative technology for evaluation of sperm DNA double-strand breaks diagnoses male factor infertility and prevents reproductive failures
Source: Sci Rep. 2023 Nov 3;13:18996. doi: 10.1038/s41598-023-46049-4 (PMC10624885; doi:10.1038/s41598-023-46049-4)
Supplement: Supplementary file 4 — Supplementary Table 3. [file 41598_2023_46049_MOESM4_ESM.docx]

**
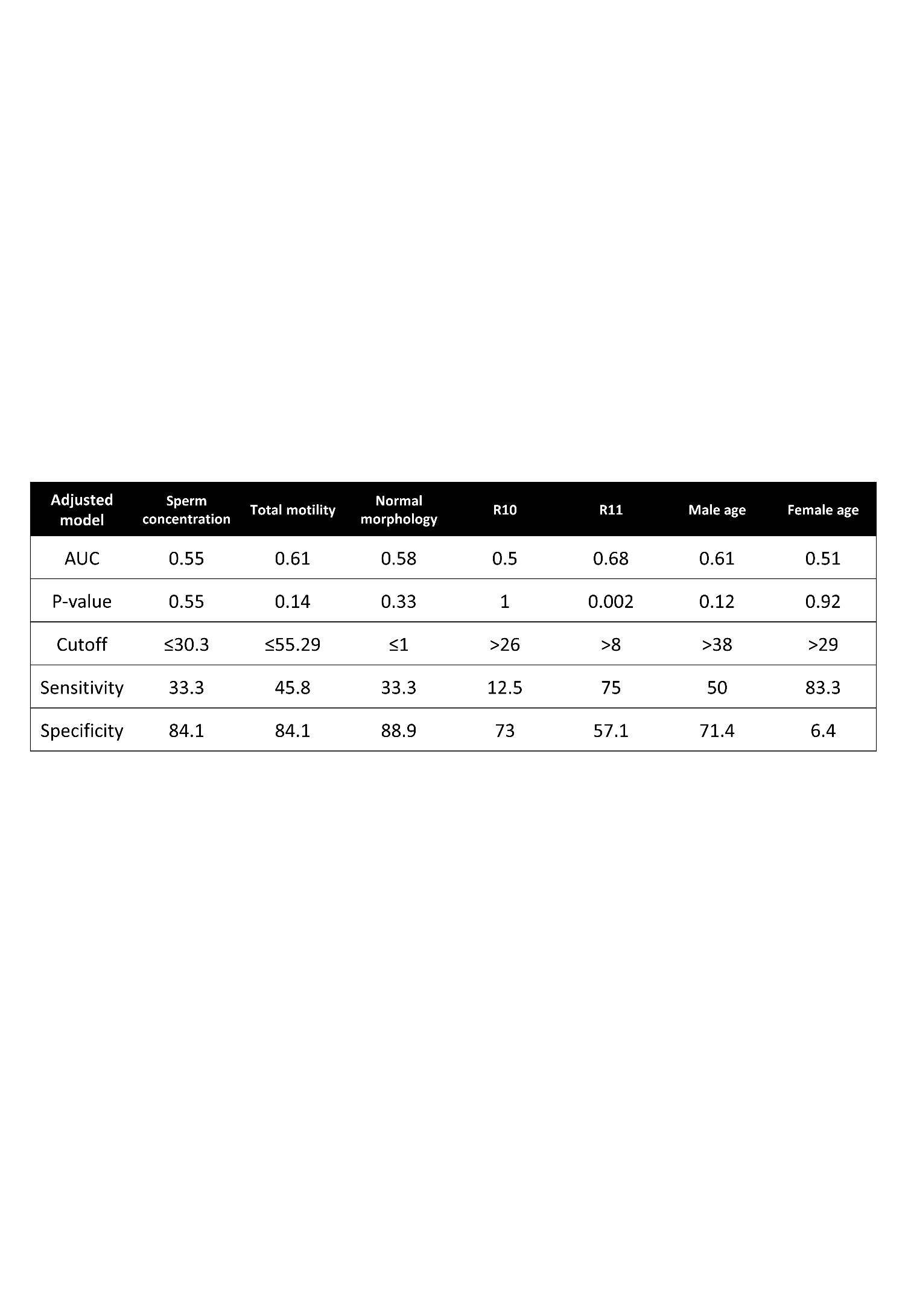
Supplementary Table S3. Performance matrix of adjusted model for high embryonic aneuploidy prediction.**

*Abbreviation: AUC (area under curve); R10 (LensHooke^®^ R10 sperm chromatin dispersion assay); R11 (LensHooke^®^ R10 sperm DNA fragmentation releasing assay)
